# Iron Species-Supporting Hydrophobic and Nonswellable Polytetrafluoroethylene/Poly(acrylic acid-co-hydroxyethyl methacrylate) Composite Fiber and Its Stable Catalytic Activity for Methylene Blue Oxidative Decolorization

**DOI:** 10.3390/polym13101570

**Published:** 2021-05-13

**Authors:** Naiku Xu, Mengru Ren

**Affiliations:** State Key Laboratory of Separation Membranes and Membrane Processes, College of Material Science and Engineering, Tiangong University, Tianjin 300387, China; rose1025174@163.com

**Keywords:** composite fiber, heterogeneous Fenton catalyst, water-swelling resistance, hydrophobicity, methylene blue oxidative decolorization

## Abstract

Polytetrafluoroethylene emulsion was ultrasonically mixed with an extremely spinnable poly(acrylic acid-co-hydroxyethyl methacrylate) solution to get a dispersion with good spinnability, and the obtained dispersion was then wet-spun into water-swellable fiber. Crosslinking agents and iron species were simultaneously introduced into the water-swellable fiber through simple impregnation and water swelling. A composite fiber with Fenton reaction-catalyzing function was then fabricated by sequentially conducting crosslinking and sintering treatment. Due to crosslinking-induced good resistance to water swelling and PTFE component-induced hydrophobicity, the composite fiber showed a highly stable activity to catalyze H_2_O_2_ to oxidatively decolorize methylene blue (MB). Within nine cycles, the composite fiber could decolorize more than 90% of MB within one minute in the presence of H_2_O_2_ and did not show any attenuation in MB decolorization efficiency. The composite fiber still could reduce the total organic carbon of MB aqueous solution from 18.3 to 10.3 mg/L when used for the ninth time. Therefore, it is believable that the prepared fiber has good and broad application prospects in the field of dye wastewater treatment.

## 1. Introduction

In recent years, the textile industry has ushered in an opportunity to develop new products due to the increasing demand for high-performance and special textiles in different countries of the world. However, the textile industry is water-intensive throughout its many processes and can simultaneously produce considerable amounts of wastewater in the processes of printing and dyeing. The wastewater generated during printing and dyeing contains a variety of organic dyes, and most of the dyes are toxic, carcinogenic, and non-biodegradable, which seriously threatens water ecosystems and human health [1,2,3]. As a result, it is absolutely necessary to decontaminate printing and dyeing wastewater.

Advanced oxidation processes (AOPs) can, in situ, produce highly reactive hydroxyl radicals (·OH). The reactive species is an extremely strong oxidant that can nonselectively oxidize most compounds present in water into small inorganic molecules such as carbon dioxide and water, and the whole oxidation process shows advantages such as short reaction time and high oxidation efficiency [4,5,6,7]. As a result of these advantages, AOPs are considered to be a more promising method for the disposal of dye wastewater. Among them, Fenton technology, which relies on the reaction between water-soluble iron salt and hydrogen peroxide (H_2_O_2_) to produce ·OH, has attracted the attention of scientists and engineers due to its convenient operability, mild reaction condition, and low cost. However, the homogeneous Fenton process works within a narrow pH range and consumes large amounts of H_2_O_2_, and it simultaneously produces a large amount of iron-rich sludge [8,9,10]. These disadvantages significantly limit the large-scale application of the homogeneous Fenton process. In order to solve these problems caused by the homogeneous Fenton process, more and more researchers have proposed and adopted the heterogeneous Fenton process to purify polluted water. The heterogeneous Fenton process usually uses catalysts prepared by immobilizing active iron-based components onto carriers through ion exchange and/or chemical coordination to catalyze H_2_O_2_ to produce ·OH. The heterogeneous Fenton process can effectively widen the applicable pH range, minimize the H_2_O_2_ consumption, and decrease the production of iron-rich sludge. Materials such as carbon materials [11], diatomite [12], a molecular sieve [13], and fiber [14] are commonly used as carriers. Among these carriers, fiber has the advantages including low cost, low density, high strength, good elasticity, excellent processability, and convenient applicability; thus, the heterogeneous Fenton catalyst prepared with fiber as a carrier is becoming a new research hotspot.

Polytetrafluoroethylene (PTFE) is a versatile high-performance fluoropolymer made up of carbon and fluorine atoms. The size of fluorine atom allows the formation of a uniform and continuous sheath around carbon–carbon bonds, which can protect these carbon–carbon bonds from chemical attack and impart excellent properties such as exceptional chemical resistance and stability, good heat and low temperature resistance, outstanding resistance to light, UV, and weathering, good flexibility, and high hydrophobicity and oleophobicity to PTFE molecules [15,16]. As a result of its exceptional stability in a harsh environment, PTFE fiber is considered to be an extremely competent carrier for the preparation of a heterogeneous Fenton catalyst. In addition, the outstanding hydrophobicity of PTFE can make the catalyst prepared with PTFE fiber as a carrier more hydrophobic. Consequently, separating the catalyst from the treated water and reusing the catalyst become very convenient. However, the sheath around carbon–carbon bonds formed by fluorine atoms repels almost all other materials; thus, other materials cannot be bonded to PTFE molecules. In this sense, PTFE fiber is unable to immobilize active iron-based components firmly. Poly(acrylic acid-co-hydroxyethyl methacrylate) (P(AA-co-HEMA)) usually has good spinnability [17,18,19], and its fiber can easily bind active iron-based components by the chemical coordination of carboxyl/hydroxyl groups with iron ions. However, P(AA-co-HEMA) has excellent hydrophilicity and thus shows very weak resistance to water swelling, which greatly restricts its application as a carrier. In this instance, compared with single PTFE fiber or P(AA-co-HEMA) fiber, the fiber prepared by combining PTFE with P(AA-co-HEMA) is a more competitive carrier for the preparation of a heterogeneous Fenton catalyst. However, there are still no reports about a heterogeneous Fenton catalyst prepared by using PTFE/P(AA-co-HEMA) composite fiber as a carrier. In this case, using PTFE/P(AA-co-HEMA) composite fiber as a carrier to prepare heterogeneous Fenton catalysts is novel.

In this work, we first used suspension spinning to resolve the difficulty of spinning PTFE into fiber. P(AA-co-HEMA) was dissolved in an extremely dilute aqueous solution of sodium hydroxide to prepare a spinning solution, and PTFE emulsion was then added into the spinning solution. The mixture of P(AA-co-HEMA) solution and PTFE emulsion was ultrasonically dispersed to prepare a dispersion, in which PTFE fine particles were well suspended. The prepared dispersion was subsequently wet-spun into fibers using dilute sulfuric acid as a coagulant. Then, we chose a special liquid mixture to post-treat the formed fiber to finish the simultaneous loading of iron ions and a crosslinking agent. The wet-spun fiber was used as a carrier to immobilize iron ions by simple impregnation. During impregnation, erythritol, that was used as a crosslinking agent, was simultaneously introduced into the fiber. Heat treatment was subsequently adopted to initiate the esterification reaction between P(AA-co-HEMA) and erythritol to crosslink the wet-spun fiber, giving the wet-spun fiber good thermal resistance. The crosslinked fiber was finally sintered in a muffle furnace to fuse PTFE fine particles into a new continuous phase to obtain a composite fiber with Fenton reaction-catalyzing function. Crosslinking could help enhance the thermal stability of P(AA-co-HEMA); thus, P(AA-co-HEMA) did not decompose during sintering. In this case, P(AA-co-HEMA) could help the obtained fiber to lock active iron-based components firmly, and the continuous PTFE phase could impart good environmental resistance to the obtained fiber. As a result, the obtained fiber had an excellent ability to catalyze H_2_O_2_ to oxidatively decolorize dyes. Consequently, this work is expected to advance the development and application of PTFE-based fibers and to shed light on the preparation of novel heterogeneous Fenton catalysts.

## 2. Experimental

### 2.1. Materials

Polytetrafluoroethylene (PTFE) emulsion with a solid content of 60 wt% was supplied by Shandong Dongyue Polymer Material Co., Ltd., China. Acrylic acid (AA) was purchased from Tianjin Guangfu Fine Chemical Research Institute, China. Hydroxyethyl methacrylate (HEMA) was purchased from Tianjin Chemical Reagent Research Institute, China. Benzoyl peroxide (BPO) was purchased from Tianjin Yingdaxigui Chemical Reagent Factory, China. Erythritol was purchased from Tianjin Heowns Biochemical Technology Co., Ltd., China. Sodium hydroxide (NaOH), ferrous chloride tetrahydrate (FeCl_2_·4H_2_O), concentrated sulfuric acid (H_2_SO_4_, 98%) and hydrogen peroxide (H_2_O_2_) aqueous solution (30 wt%) were all bought from Tianjin Fengchuan Chemical Reagent Technologies Co., Ltd., Tianjin, China. Methylene blue (MB), hydroxylammonium chloride (HONH_2_Cl) and 1,10-Phenanthroline monohydrate (C_12_H_8_N_2_·H_2_O) were purchased from Tianjin Tianxin Fine Chemical Development Center, China. 5,5-Dimethyl-1-Pyrroline N-oxide (DMPO) was purchased from Shanghai Aladdin Biochemical Technology Co., Ltd., China. The water used in this work was deionized water, which was offered by Tianjin Lanyang Industrial Water Plant, Tianjin, China.

### 2.2. Polymer Synthesis

The copolymer of AA and HEMA (P(AA-co-HEMA)) was synthesized as per the published papers [18,19], describing a detailed characterization of this copolymer. In brief, a solution was first prepared by dissolving 0.0833 g BPO into 16.6667 g mixed liquid of AA and HEMA at room temperature. Fifty grams of H_2_O was added into the prepared solution and then transferred into a 250 mL four-necked flask equipped with a stirrer and a reflux condenser. The mixture in the flask was stirred to react at 85 °C for 30 min under nitrogen atmosphere, and another solution prepared by dissolving 0.1667 g BPO in 33.3333 g AA at room temperature was dropwise dripped into the flask during this for 30 min. The mixture in the flask with a mass ratio of AA to HEMA of 9:1 was then stirred at 85 °C to react for another two hours under nitrogen atmosphere. The resulting product was separated from the water using vacuum filtration, followed by hot and cool water washing, vacuum oven desiccation, and high-speed pulverization. Finally, powdered polymer was obtained.

### 2.3. Fiber Formation

The dry copolymer powder with a mass of 1.5 g was added into 30 g NaOH aqueous solution with a mass concentration of 3% and left at room temperature for about 12 h. The mixture was then magnetically stirred at 80 °C until the copolymer completely dissolved. The solution was cooled down to room temperature. Five grams of PTFE emulsion was then added in the solution and vigorously stirred for about 30 min at room temperature, followed by ultrasonic dispersion for 1 h and vacuum degassing for 30 min. The obtained dispersion was then pumped into a one-hole spinneret with a diameter of 0.42 mm at a speed of 0.41 mL/min. The stream extruded from the spinneret was immediately coagulated in a H_2_SO_4_ aqueous solution with a volume concentration of 30%, and as-spun fiber was acquired after water washing. The as-spun fiber prepared with P(AA-co-HEMA) as raw material was labeled as F-1, and the one prepared from the mixture of P(AA-co-HEMA) and PTFE was referred to as F-2. In addition, the fibers obtained by drying the fibers F-1 and F-2 in a 60 °C vacuum oven for 2.5 h were correspondingly labeled as O-1 and O-2.

### 2.4. Catalyst Preparation

A solution, which was prepared by mixing 30 g concentrated H_2_SO_4_, 16.83 g FeCl_2_·4H_2_O, and 120 g deionized water, was labeled as I_1_, and another solution composed of 30 g concentrated H_2_SO_4_, 16.83 g FeCl_2_·4H_2_O, 20 g erythritol, and 100 g deionized water was referred to as I_2_. The as-spun fiber F-1 was cut into 3 cm long staple fibers. Thirty-five staple fibers were then immersed into solution I_1_. After 2 h, the staple fibers were separated from solution I_1_, followed by vacuum drying for 2.5 h at 60 °C, and the obtained catalyst was named C_1_-1. When solution I_2_ was used, the above process was repeated once again to prepared catalyst C_2_-1, except that vacuum drying for 2.5 h at 60 °C was replaced by vacuum crosslinking for 30 min at 200 °C. In order to confirm whether a crosslinking reaction happened or not, the control sample J-1 was also prepared by immersing 35 staple fibers (F-1) into a solution composed of 20 g erythritol and 146.83 g H_2_O for 2 h and then performing vacuum crosslinking for 30 min at 200 °C. For the as-spun fiber F-2, 35 staple fibers (F-2) were immersed in solution I_2_. After 2 h, the staple fibers were separated from the solution, followed by vacuum crosslinking for 30 min at 200 °C, and the obtained catalyst was named C_2_-2. After catalyst C_2_-2 was sintered in a 380 °C muffle furnace for 7 min, the resulting catalyst was referred to as C_2_-2S. In order to analyze the influence of iron ions on the structural features of the fiber, control sample J-2 was prepared as per the preparation procedure and parameters of C_2_-2S, except that the impregnation solution did not contain FeCl_2_·4H_2_O.

The fiber samples prepared as described in Section 2.3 and Section 2.4 are summarized in Table 1.

### 2.5. Measurement and Characterization

#### 2.5.1. Morphology

The cross-section of the fiber sample was obtained by performing brittle fracture in liquid nitrogen, and the surface and cross-section of the fiber sample were subsequently coated with gold through electrodeposition. A Gemini SEM500 thermal field emission scanning electron microscope (FESEM) (Carl Zeiss, Germany) was then used to observe the morphological features of the fiber sample under an accelerating voltage of 10.0 kV.

#### 2.5.2. Chemical Group

The chemical group of the fiber sample was analyzed by using a Nicolet iS50 Fourier transform infrared spectrometer (FTIR) (Thermofisher Scientific Company, America) at a light incident angle of 45° and a resolution of 0.09 cm^−1^.

#### 2.5.3. Heat Resistance

The thermal property of the fiber sample was analyzed by heating the fiber sample from 20 to 800 °C in a STA449F3 thermogravimetric analyzer (TGA) (Netzsch Group, Erlangen, Germany) at a heating rate of 10 °C/min under nitrogen atmosphere.

#### 2.5.4. Water Contact Angle

The static water contact angle of the fiber sample was measured using a liquid-drop method. The fiber sample was first completely dried in a 45 °C vacuum oven, and its water contact angle was then measured at room temperature by using a JC200DM contact angle tester equipped with a high-resolution CCD camera (Shanghai Zhongchen Digital Technology Equipment Co., Ltd., Shanghai, China).

#### 2.5.5. Surface Element

The surface element of the fiber sample was analyzed by using a K-alpha X-ray photoelectron spectrometer (XPS) (Thermofisher Scientific Company, Waltham, America) with monochromatized Al Kα as a radiation resource, and its spot size, pass energy, and energy step size were 400 μm, 50.0 eV, and 0.100 eV, respectively. The analyzer was operated under the mode of constant analyzer energy (CAE).

#### 2.5.6. Aggregate Structure

The aggregate structure of the fiber sample was researched by using a D8 Advance X-ray diffractometer (XRD) (Bruker, Karlsruhe, Germany). The diffractometer with a copper cathode was operated in reflection mode at a Cu K_α1_ wavelength of 1.5406 Å, a voltage of 40 kV, and a current of 40 mA. The measurement was performed within a 2-theta scale of 10 to 80° at a scanning rate of 5°/min.

#### 2.5.7. Water Resistance

The fiber O-1 or J-1 with a given mass was immersed in NaOH aqueous solution with a mass concentration of 0.1% for 30 min. The fiber was then separated from the solution. After surface water was removed with filter paper, the fiber was weighed. The water absorbency was calculated as per the following equation.

(1) $$WA=\frac{m-m_{0}}{m_{0}}$$

where WA is the water absorbency, g/g; m_0_ is the mass of initial fiber; m is the mass of swollen fiber.

#### 2.5.8. Catalytic Activity

Methylene blue (MB) was chosen as a target pollutant, and the activity of the prepared fiber to catalyze H_2_O_2_ to oxidatively decontaminate MB from its aqueous solution was evaluated as per the following procedures. Ten milliliters of MB aqueous solution with a concentration of 20.0 mg/L was added into a 50 mL beaker, and 2 μL H_2_O_2_ aqueous solution with a concentration of 30 wt% was then transferred into the beaker using a pipetting gun. Ten staple fibers prepared as described in Section 2.4 were added into the beaker, and the beaker was shaken at room temperature. A TU-1810 ultraviolet–visible spectrophotometer (Beijing Purkinje General Instrument Co., Ltd., Beijing, China) was used to measure the absorbance of MB aqueous solution at a wavelength of 664 nm, and the decolorization efficiency of MB aqueous solution (DE) was calculated using Equation (2). As the decolorization efficiency reached up to 95% or the reaction time reached 90 min, the fibers were separated from MB aqueous solution. After being completely dried in a vacuum oven, the fibers were used once again following the above steps, and the reusability could be evaluated. In addition, iron ions eluted by MB aqueous solution during decolorization were quantified as per the method described in the published paper [17].

(2) $$DE=\frac{A_{0}-A_{t}}{A_{0}}\times 100$$

where DE is the decolorization efficiency, %; A_0_ is the absorbance of initial MB aqueous solution; A_t_ is the absorbance of MB aqueous solution obtained at a given decolorization time.

#### 2.5.9. Total Organic Carbon

A TOC-VCPH total organic carbon analyzer (Shimadzu Corp., Kyoto, Japan) was used to measure the content of total organic carbon in the original MB aqueous solution or MB aqueous solution treated with the fiber C_2_-2S as a catalyst in the presence of H_2_O_2_.

#### 2.5.10. Free Radical Species

Ten milliliters of deionized water was added into a 50 mL beaker, and 2 μL H_2_O_2_ aqueous solution with a concentration of 30 wt% was then transferred into the beaker using a pipetting gun. Ten staple fibers prepared as described in Section 2.4 were added into the beaker. The system was shaken to react for 1 min at room temperature. Two hundred microliters of reaction solution was then transferred into a small container, and 40 μL DMPO aqueous solution with a concentration of 0.2 mol/L was added into the container. After being mixed sufficiently, the mixed solution was transferred to a quartz capillary, and a JES-FA200 electron paramagnetic resonance (EPR) spectrometer (JEOL, Akishima, Japan) was then used to monitor the free radical species.

## 3. Results and Discussion

### 3.1. The Confirmation of Crosslinking

**Figure 1:**
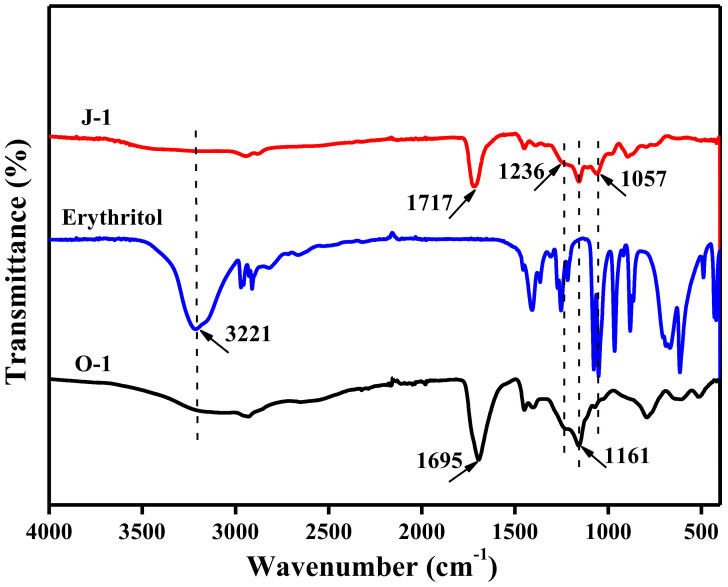
FTIR spectra of the prepared fibers O-1 and J-1; fiber O-1 is defined in Section 2.3, and fiber J-1 is described in Section 2.4.

**Figure 2:**
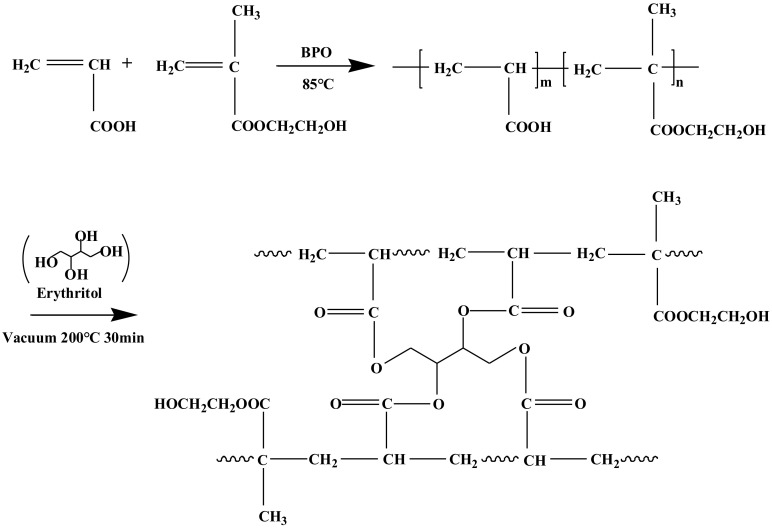
The reactions induced by heat treatment.

**Figure 3:**
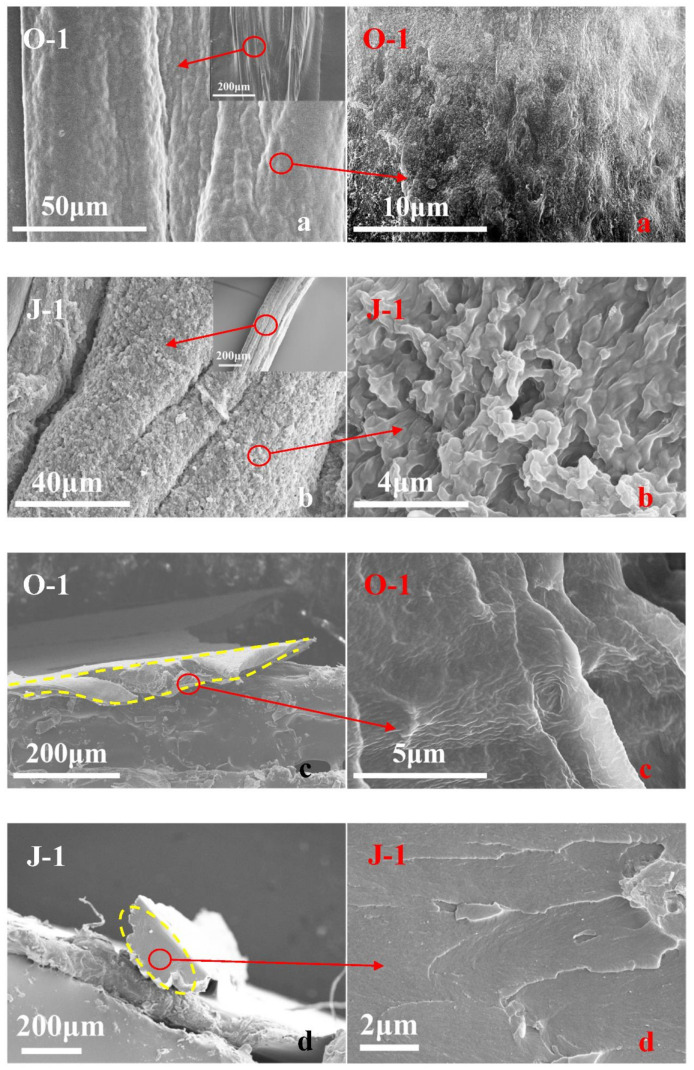
SEM images of the fibers obtained before and after crosslinking treatment; fiber O-1 is defined in Section 2.3, and fiber J-1 is described in Section 2.4; the surface of fiber O-1 (**a**), the surface of fiber J-1 (**b**), the cross section of fiber O-1 (**c**) and the cross section of fiber J-1 (**d**).

The prepared fiber O-1 shows the stretching vibration peaks of O-H and C=O of the AA structural unit at 3221 [20] and 1695 cm^−1^ [21], as described in Figure 1; additionally, the FTIR curve of fiber O-1 shows a peak at 1161 cm^−1^, which is caused by the stretching vibration of C-O in C-O-C of the HEMA structural unit [22]. Erythritol has three characteristic peaks, which are located at 3221, 1236, and 1057 cm^−1^, corresponding to the stretching vibration of O-H [20], the in-plane bending vibration of O-H [23], and the stretching vibration of C-O in C-O-H [22]. In contrast to fiber O-1, fiber J-1 shows the stretching vibration peak of C-O in C-O-H at 1057 cm^−1^, indicating that erythritol is introduced into fiber J-1. However, fiber J-1 does not show the vibration peak of O-H as clearly as erythritol or fiber O-1 does, and the stretching vibration peak of C=O of fiber J-1 shifts to 1717 cm^−1^, which is usually caused by the stretching vibration of C=O of the ester group [24]. This indicates that vacuum heat treatment can induce the esterification reaction between erythritol and P(AA-co-HEMA) to lead to crosslinking, as shown in Figure 2. Figure 3a shows that the fiber obtained before crosslinking (O-1) has a smooth surface, and its body is highly dense, as shown in Figure 3c. Furthermore, fiber O-1 is very flat, as shown in Figure 3c, which implies that P(AA-co-HEMA) has a very weak ability to support itself to form fiber when it is wet or in a swelling state. Figure 3b shows that crosslinked fiber J-1 has a much rougher surface than uncrosslinked fiber O-1, and Figure 3d shows that crosslinked fiber J-1 has a highly dense body like uncrosslinked fiber O-1. However, crosslinked fiber J-1 is endowed with a nearly elliptical shape, as shown in Figure 3d. This phenomenon makes us believe that crosslinking is an effective approach to improve the self-supportability of P(AA-co-HEMA). The improved self-supportability is very useful for fiber formation and application. As a result, it is expected that the transformation of micro- and macrostructures induced by crosslinking treatment can positively enhance the application performance of the prepared fiber.

### 3.2. The Significance of Crosslinking

**Figure 4:**
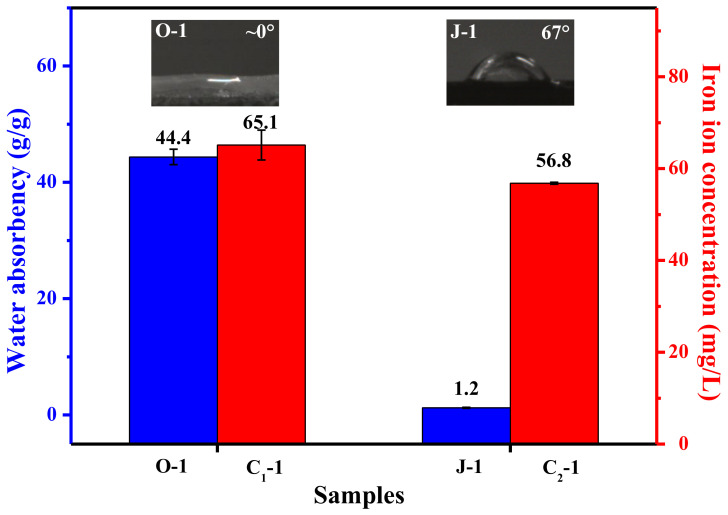
Water absorbency of the fiber sample and the concentration of iron ions leached into the treated MB aqueous solution; fiber O-1 is defined in Section 2.3, and the fibers J-1, C_1_-1, and C_2_-1 are described in Section 2.4; iron ion concentration here refers to the average concentration of iron ions eluted into the MB aqueous solutions obtained during the first three cycles; the inset pictures show the water contact angle of the fiber sample.

**Figure 5:**
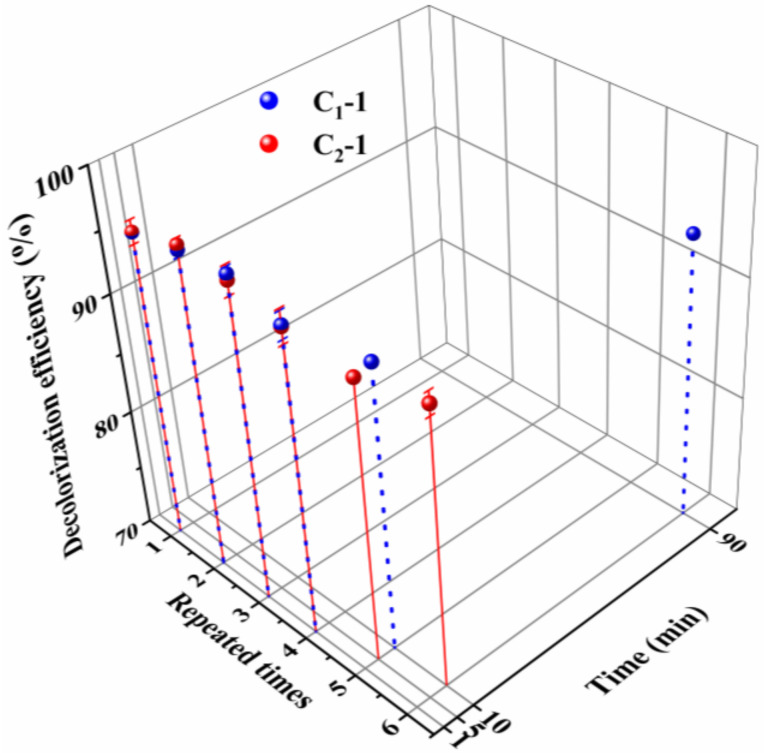
The performance of the fiber to decolorize MB aqueous solution during its repeated application; the fibers C_1_-1 and C_2_-1 are described in Section 2.4.

**Figure 6:**
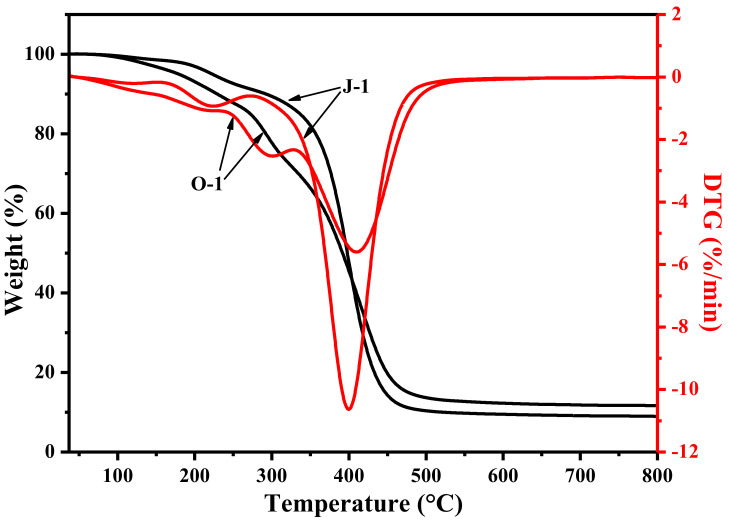
The effect of crosslinking on the heat resistance of the fiber; fiber O-1 is defined in Section 2.3, and fiber J-1 is described in Section 2.4.

The reusability of an iron species-supporting catalyst is determined by the amount of iron ions leached during application, and the amount of iron ions leaching out is dependent on the catalyst’s hydrophilicity and swellability. In principle, if the catalyst is extremely hydrophilic and swellable, it will generally show poor reusability due to high leaching out amount of iron ions. Figure 4 shows that the crosslinked fiber exhibits a higher water contact angle than the uncrosslinked fiber; additionally, the crosslinked fiber has a much lower water absorption capability than the uncrosslinked fiber. This result demonstrates that crosslinking can effectively reduce hydrophilicity and swellability. Due to the decreased hydrophilicity and swellability, the catalyst prepared by using the crosslinked fiber as a carrier presents lower leaching out amount of iron ions than its counterpart prepared with the uncrosslinked fiber as a carrier. Thereby, compared with catalyst C_1_-1, catalyst C_2_-1 is expected to show better reusability. In fact, when the catalysts C_2_-1 and C_1_-1 are repeatedly used to decolorize fresh MB aqueous solution, a distinguishable difference can be found, as shown in Figure 5. When they are used for the fifth time, catalyst C_2_-1 takes only 5 min to decolorize 90% of MB, but catalyst C_1_-1 needs 10 min to reach a decolorization efficiency of 90%. For the sixth use, catalyst C_2_-1 just takes 10 min to decolorize 90% of MB, but catalyst C_1_-1 needs 90 min to reach an equivalent decolorization efficiency. In this case, crosslinking is considered to be a viable and effective way to improve the fibrous catalyst’s application performance. In addition, Figure 6 shows that the crosslinked fiber has lower weight loss at temperatures below 400 °C and a slower weight loss rate at temperatures below 360 °C than its counterpart, indicating that the crosslinked fiber possesses better thermal stability than the uncrosslinked fiber. During sintering, the enhanced heat resistance can facilitate the successful preparation of PTFE/P(AA-co-HEMA) composite fiber by preventing the decomposition of P(AA-co-HEMA).

### 3.3. The Role of PTFE

**Figure 7:**
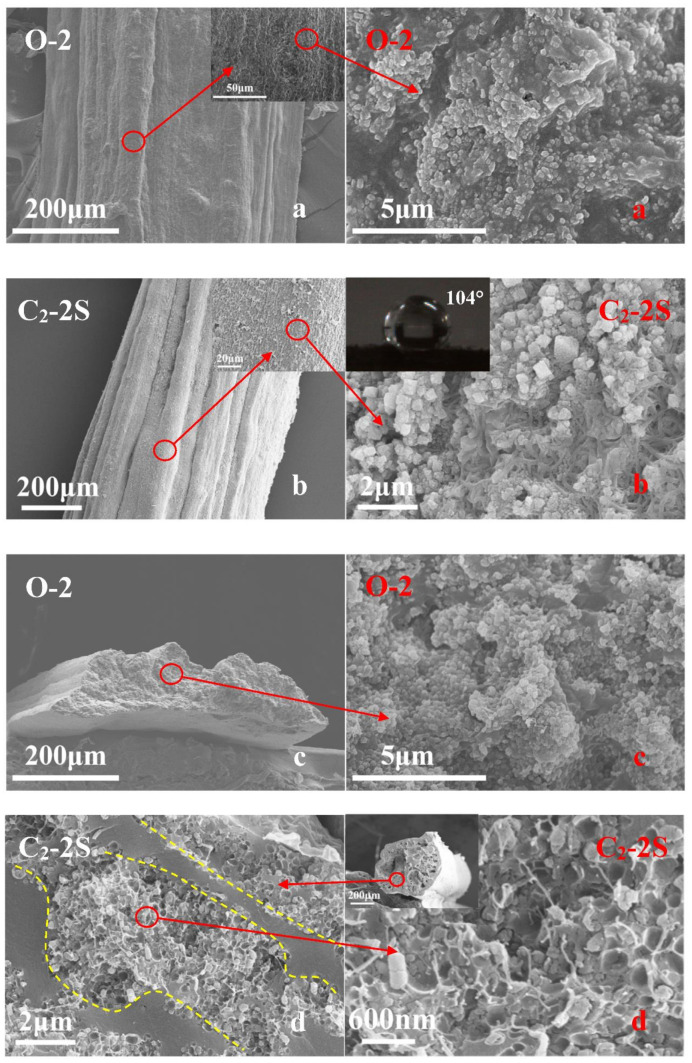
SEM images of the fibers obtained before and after sintering; fiber O-2 is defined in Section 2.3, and fiber C_2_-2S is described in Section 2.4; the surface of fiber O-2 (**a**), the surface of fiber C_2_-2S (**b**), the cross section of fiber O-2 (**c**) and the cross section of fiber C_2_-2S (**d**).

**Figure 8:**
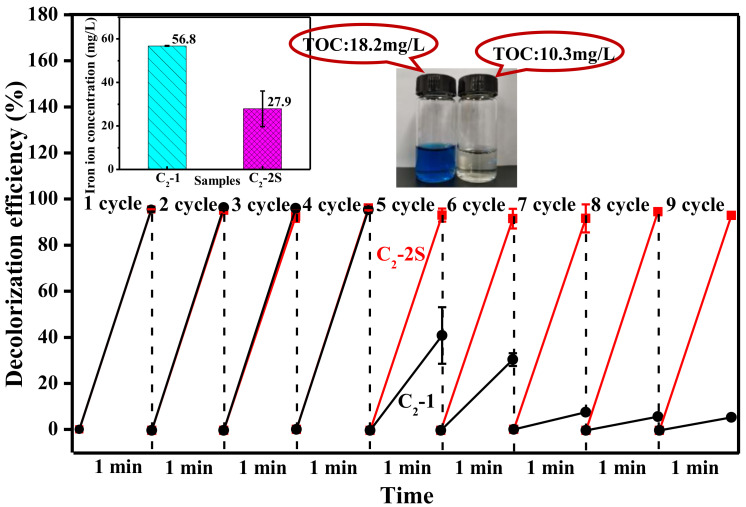
Decolorization efficiency in the process of reusing the fibers C_2_-1 and C_2_-2S to decolorize fresh MB aqueous solution; the fibers C_2_-1 and C_2_-2S are defined in Section 2.4; the graph inset in the upper left corner shows the average concentration of iron ions eluted into the MB aqueous solutions obtained during the first three cycles; the picture inset in the upper right corner represents the TOC values of fresh MB aqueous solution and MB aqueous solution obtained at a treatment time of 1 min during the ninth cycle of fiber C_2_-2S.

Fiber O-2 has a smooth surface and a dense body similar to fiber O-1, and it also shows a very flat shape, as shown in Figure 7a,c. However, the magnified images in Figure 7a,c reveal that fiber O-2 has fine PTFE particles on its surface and inside, implying that PTFE has been introduced into fiber O-2. When these treatment processes, such as iron ion loading, crosslinking, and sintering are conducted, fiber O-2 is converted into fiber C_2_-2S. Figure 7b shows that fiber C_2_-2S has a rougher surface than fiber O-2, and its magnified image reveals that PTFE particles are fused into microfibrils, and a lot of fine iron-based particles are generated during the preparation of fiber C_2_-2S. Due to the rougher surface caused by these PTFE microfibrils and fine iron-based particles, fiber C_2_-2S has a water contact angle as high as 104°, as shown in Figure 7b. Figure 7d shows that the cross-section of fiber C_2_-2S shows a two-region interlocking structure, in which a highly dense phase and relatively loose phase can be found. According to the structural morphology of the fiber J-1, which is shown in Figure 3d, the dense phase mentioned here is formed by crosslinked P(AA-co-HEMA), and the loose phase is related to PTFE. The magnified image in Figure 7d can help us find PTFE microfibrils and the small pits formed due to the removal of fine iron-based particles during brittle fracture. In this instance, the existence of PTFE microfibrils not only makes fiber C_2_-2S more hydrophobic but also imparts an interlocking structure to fiber C_2_-2S; thus, fiber C_2_-2S is thought to be more reusable than fiber C_2_-1. When fiber C_2_-2S is used to decolorize MB aqueous solution, it does not show any loss in decolorization efficiency within nine cycles, and more than 90% of MB can be decolorized within one minute, as shown in Figure 8. At the ninth cycle, MB aqueous solution obtained after decolorization has a TOC value of 10.3 mg/L, which is much lower than 18.2 mg/L of fresh MB aqueous solution, indicating that MB is partially oxidized into CO_2_ and H_2_O during decolorization. In contrast to fiber C_2_-2S, fiber C_2_-1 shows an obvious attenuation of decolorization efficiency during its reuse. Therefore, fiber C_2_-2S is considerably more reusable than fiber C_2_-1. In addition to better reusability, fiber C_2_-2S also exhibits a lower level of iron ion leaching out than fiber C_2_-1 under the same conditions, as shown in the graph inset in the upper left corner of Figure 8. Hence, PTFE plays an important role in improving the fiber’s reusability in decolorizing MB aqueous solution.

### 3.4. The Decolorization Mechanism

**Figure 9:**
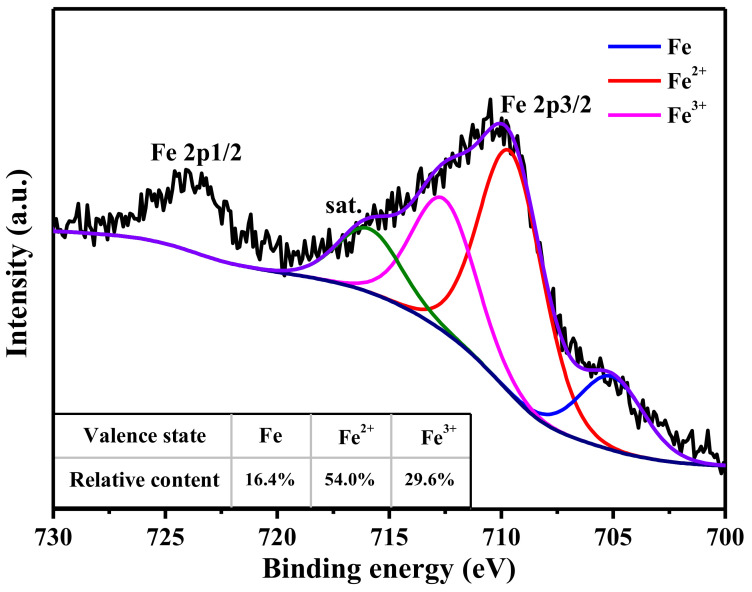
Fe2p spectra of fiber C_2_-2S; fiber C_2_-2S is defined in Section 2.4.

**Figure 10:**
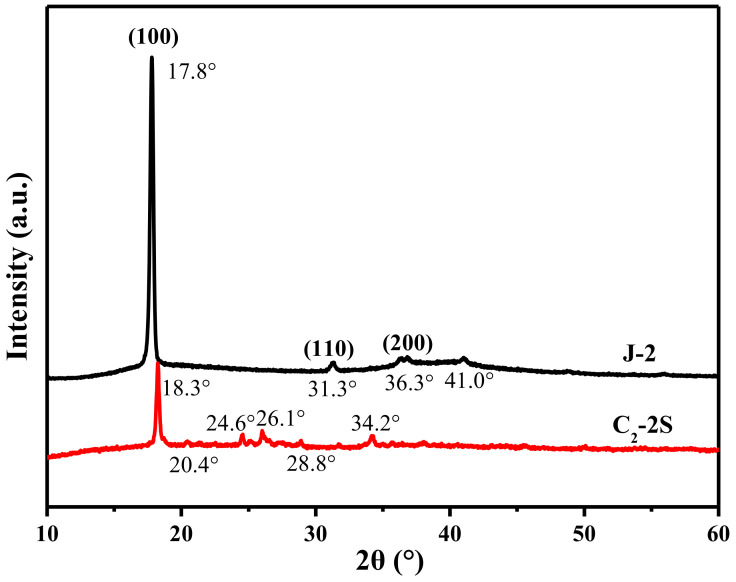
XRD patterns of the fibers J-2 and C_2_-2S; the fibers J-2 and C_2_-2S are defined in Section 2.4.

**Figure 11:**
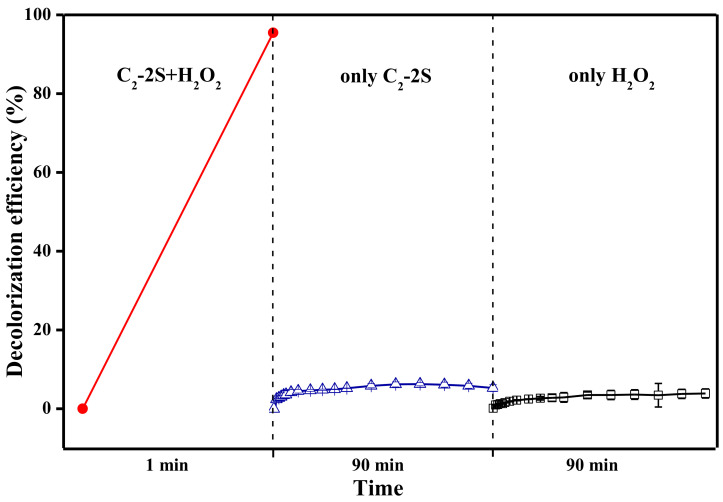
MB decolorization efficiency of different systems; the left shows the system including MB aqueous solution, H_2_O_2_, and fiber C_2_-2S; the middle shows the system including MB aqueous solution and fiber C_2_-2S; the right shows the system including MB aqueous solution and H_2_O_2_; fiber C_2_-2S is defined in Section 2.4.

**Figure 12:**
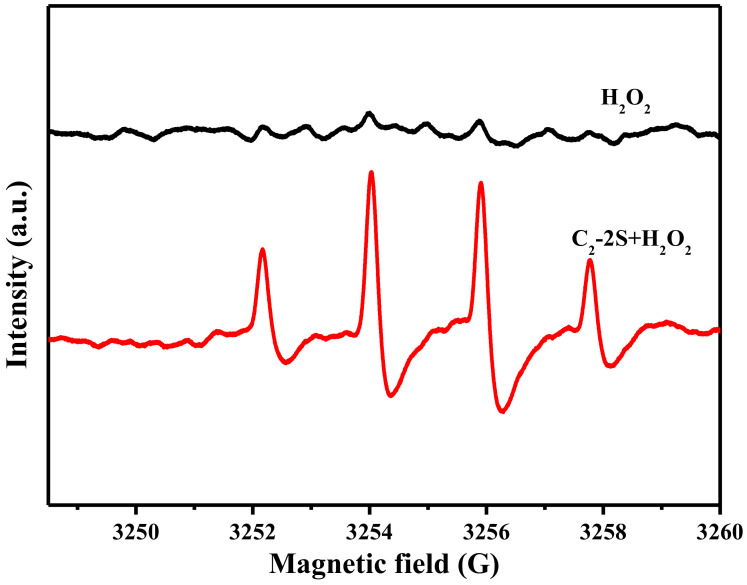
EPR spectra of deionized water in the presence of H_2_O_2_ or the combination of fiber C_2_-2S and H_2_O_2_; fiber C_2_-2S is defined in Section 2.4.

Fe 2p3/2 peak can be deconvoluted into four components, which are located at 705.1, 709.5, 712.5, and 715.9 eV, as shown in Figure 9. These components correspond to metallic Fe, Fe^2+^, Fe^3+^, and satellite peak [25,26]. The table in Figure 9 describes the relative content of metallic Fe, Fe^2+^, and Fe^3+^, which is calculated based on the area of the deconvoluted peaks. The XRD curve of fiber J-2, as shown in Figure 10, shows three diffraction peaks at 17.8, 31.3, and 36.3°, corresponding to the (100), (110), and (200) lattice planes of PTFE [27]. The XRD curve of fiber J-2 also shows a small peak at 41.0°, which is caused by the turbostratic structure of disordered carbon [28,29], which implies that sintering treatment can result in the production of a small amount of carbon. The XRD curve of fiber C_2_-2S shows six diffraction peaks at 18.3, 20.4, 24.6, 26.1, 28.8, and 34.2°. The diffraction peak of the PTFE (100) lattice plane increases to 18.3°, which indicates that an interaction occurs between iron species and PTFE molecules during sintering and the interaction narrows the interlayer space of the PTFE (100) lattice plane [30]. The interaction between iron species and PTFE molecules is one of the reasons why fiber C_2_-2S shows low leaching out of iron ions during application. A low level of leaching out of iron ion ensures that fiber C_2_-2S is reusable when decolorizing MB aqueous solution. According to the related literature, the peaks at 20.4 and 26.1° are caused by FeOOH [31,32]; the peak at 24.6° by Fe_2_O_3_ [33]; the peak at 28.8° by FeO [34]; the peak at 34.2° by Fe_3_O_4_ [35]. This result demonstrates that the iron species supported by PTFE/P(AA-co-HEMA) composite fiber is mainly composed of the iron oxides mentioned above. These oxides only involve divalent and/or trivalent iron, which is in accordance with the XPS result. However, the XRD curve of fiber C_2_-2S in Figure 10 does not show a diffraction peak at 41.0°, which means that fiber C_2_-2S does not contain the disordered carbon mentioned above. Additionally, the XPS results in Figure 9 reveal that the iron species contains metallic Fe. It is thus deduced that the reduction of partial iron oxide to metallic Fe at a high temperature consumes the disordered carbon generated during sintering. Figure 11 shows that only fiber C_2_-2S containing the aforementioned iron species cannot remove MB from water; additionally, H_2_O_2_ is also unable to decontaminate MB aqueous solution. Nevertheless, as both fiber C_2_-2S and H_2_O_2_ are added into MB aqueous solution, MB decolorization is accomplished within 1 min, and decolorization efficiency reaches up to 95.5%. In addition, the TOC value of the MB aqueous solution treated in the presence of fiber C_2_-2S and H_2_O_2_ decreases significantly, as shown in Figure 8. Thereby, fiber C_2_-2S has the ability to catalyze H_2_O_2_ to produce species with strong oxidizability, which can lead to decolorization by oxidizing MB into CO_2_, H_2_O, and colorless matter. As displayed in Figure 12, no signal can be detected for the system composed of deionized water and H_2_O_2_. When fiber C_2_-2S is added into the deionized water containing H_2_O_2_, a quartet signal of the typical adduct of DMPO and hydroxyl radical (·OH) with a relative intensity of 1:2:2:1 is clearly observed [36,37]. Thereby, ·OH is the oxidative species that is produced in the presence of fiber C_2_-2S and H_2_O_2_. As a result of the abovementioned phenomena, the Fenton route is adopted to illustrate the decolorization mechanism of MB, and the corresponding reactions are listed as follows. Divalent iron supported by fiber C_2_-2S first reacts with H_2_O_2_ to produce ·OH (Equation (3)). Trivalent iron is simultaneously reduced to Fe^2+^ (Equation (4)), and the HO_2_· generated during the reduction of trivalent iron can further react with trivalent iron to produce divalent iron (Equation (5)). The generated H^+^ can also etch metallic Fe to result in the generation of divalent iron (Equation (6)). This newly generated divalent iron can be included in Equation (3) once again. The produced ·OH oxidizes MB to cause decolorization (Equation (7)). In general, Equations (4) and (5) are the steps that can limit the reaction rate, which plays an important role in controlling decolorization efficiency and reaction time [38,39]. However, fiber C_2_-2S is rich in divalent iron, and the metallic Fe supported by fiber C_2_-2S also can convert into divalent iron through Equation (6). Divalent iron can quickly promote the formation of ·OH [40]. Consequently, the catalyst prepared in this work can achieve a high decolorization efficiency within a short time during its cycling use.

(3) $${\mathrm{Fe}}^{2+}+H_{2}O_{2}\to {\mathrm{Fe}}^{3+}+•\mathrm{OH}+{\mathrm{OH}}^{{}_{-}}$$

(4) $${\mathrm{Fe}}^{3+}+H_{2}O_{2}\to {\mathrm{Fe}}^{2+}+H^{+}+{\mathrm{HO}}_{2}•$$

(5) $${\mathrm{Fe}}^{3+}+{\mathrm{HO}}_{2}•\to {\mathrm{Fe}}^{2+}+H^{+}+O_{2}$$

(6) $$\mathrm{Fe}+{2H}^{+}\to {\mathrm{Fe}}^{2+}+H_{2}$$

(7) $$\mathrm{MB}+•\mathrm{OH}\to {\mathrm{CO}}_{2}+H_{2}O+\;\mathrm{colorless}\;\mathrm{matter}$$

## 4. Conclusions

Erythritol can be used as a polyol to crosslink P(AA-co-HEMA) through multiple esterification. Crosslinking can effectively enhance the thermal stability of P(AA-co-HEMA), which ensures that sintering process can be conducted. Due to good heat resistance of P(AA-co-HEMA) caused by crosslinking, sintering P(AA-co-HEMA) fiber containing small PTFE particles and iron species can be successfully used to prepare a composite fiber based on P(AA-co-HEMA) and PTFE, which is the main objective of this work. During sintering, the iron species is firmly held in the composite fiber. In addition to a small amount of metallic Fe, the iron species supported by the composite fiber is mainly composed of iron oxide. This iron species provides the composite fiber with an excellent ability to catalyze H_2_O_2_ to produce ·OH, which gives the composite fiber application potential in decolorizing dye wastewater. Crosslinking also remarkably improves the resistance of the P(AA-co-HEMA) phase to water swelling. PTFE makes the composite fiber more hydrophobic. Furthermore, the composite fiber has a two-phase interlocking structure due to the coexistence of crosslinked P(AA-co-HEMA) and PTFE. The combining effect of water swelling resistance and hydrophobicity as well as the interlocking structure can vastly reduce the leaching out of iron ions when the composite fiber is used to decolorize MB aqueous solution, making the composite fiber considerably reusable in catalyzing H_2_O_2_ to oxidatively decolorize MB aqueous solution. In this case, these findings are expected to advance the development and application of PTFE-based fibers and to shed light on the preparation of novel heterogeneous Fenton catalysts.

## Figures and Tables

**Table 1:** The definition of fiber samples prepared as described in Section 2.3 and Section 2.4.

Raw Material	Solvent	Coagulant	As-Spun Fiber	Dry As-Spun Fiber	Treatment Reagent	Treatment Condition	Sample
P(AA-co-HEMA)	NaOH aqueous solution	H_2_SO_4_ aqueous solution	F-1	O-1	-	-	-
P(AA-co-HEMA) PTFE	NaOH aqueous solution	H_2_SO_4_ aqueous solution	F-2	O-2	-	-	-
P(AA-co-HEMA)	NaOH aqueous solution	H_2_SO_4_ aqueous solution	F-1	-	Erythritol water	200 °C, 30 min vacuum crosslinking	J-1
P(AA-co-HEMA)	NaOH aqueous solution	H_2_SO_4_ aqueous solution	F-1	-	Concentrated H_2_SO_4_ FeCl_2_·4H_2_O water	60 °C, 2.5 h vacuum drying	C_1_-1
P(AA-co-HEMA)	NaOH aqueous solution	H_2_SO_4_ aqueous solution	F-1	-	Concentrated H_2_SO_4_ FeCl_2_·4H_2_O erythritol water	200 °C, 30 min vacuum crosslinking	C_2_-1
P(AA-co-HEMA) PTFE	NaOH aqueous solution	H_2_SO_4_ aqueous solution	F-2	-	Concentrated H_2_SO_4_ FeCl_2_·4H_2_O erythritol water	200 °C, 30 min vacuum crosslinking	C_2_-2
P(AA-co-HEMA) PTFE	NaOH aqueous solution	H_2_SO_4_ aqueous solution	F-2	-	Concentrated H_2_SO_4_ FeCl_2_·4H_2_O erythritol water	200 °C, 30 min vacuum crosslinking 380 °C, 7 min sintering	C_2_-2S
P(AA-co-HEMA) PTFE	NaOH aqueous solution	H_2_SO_4_ aqueous solution	F-2	-	Concentrated H_2_SO_4_ erythritol water	200 °C, 30 min vacuum crosslinking 380 °C, 7 min sintering	J-2

## Data Availability

The raw/processed data required to reproduce these findings cannot be shared at this time as the data also forms part of an ongoing study.
